# Supplementary material for: Functional classification of tauopathy strains reveals the role of protofilament core residues
Source: Sci Adv. 2025 Jan 22;11(4):eadp5978. doi: 10.1126/sciadv.adp5978 (PMC11753436; doi:10.1126/sciadv.adp5978)
Supplement: Supplementary file 1 — Supplementary Text Figs. S1 to S9 Tables S1 to S3 Legend for movie S1 Legend for data S1 [file sciadv.adp5978_sm.pdf]

Science Advances
AAAS

Supplementary Materials for

Functional classification of tauopathy strains reveals the role of protofilament core residues

Jaime Vaquer-Alicea *et al.*

Corresponding author: Marc I. Diamond, marc.diamond@utsouthwestern.edu

Sci. Adv. **11**, eadp5978 (2025)
DOI: 10.1126/sciadv.adp5978

The PDF file includes:

Supplementary Text
Figs. S1 to S9
Tables S1 to S3
Legend for movie S1
Legend for data S1

Other Supplementary Material for this manuscript includes the following:

Movie S1
Data S1

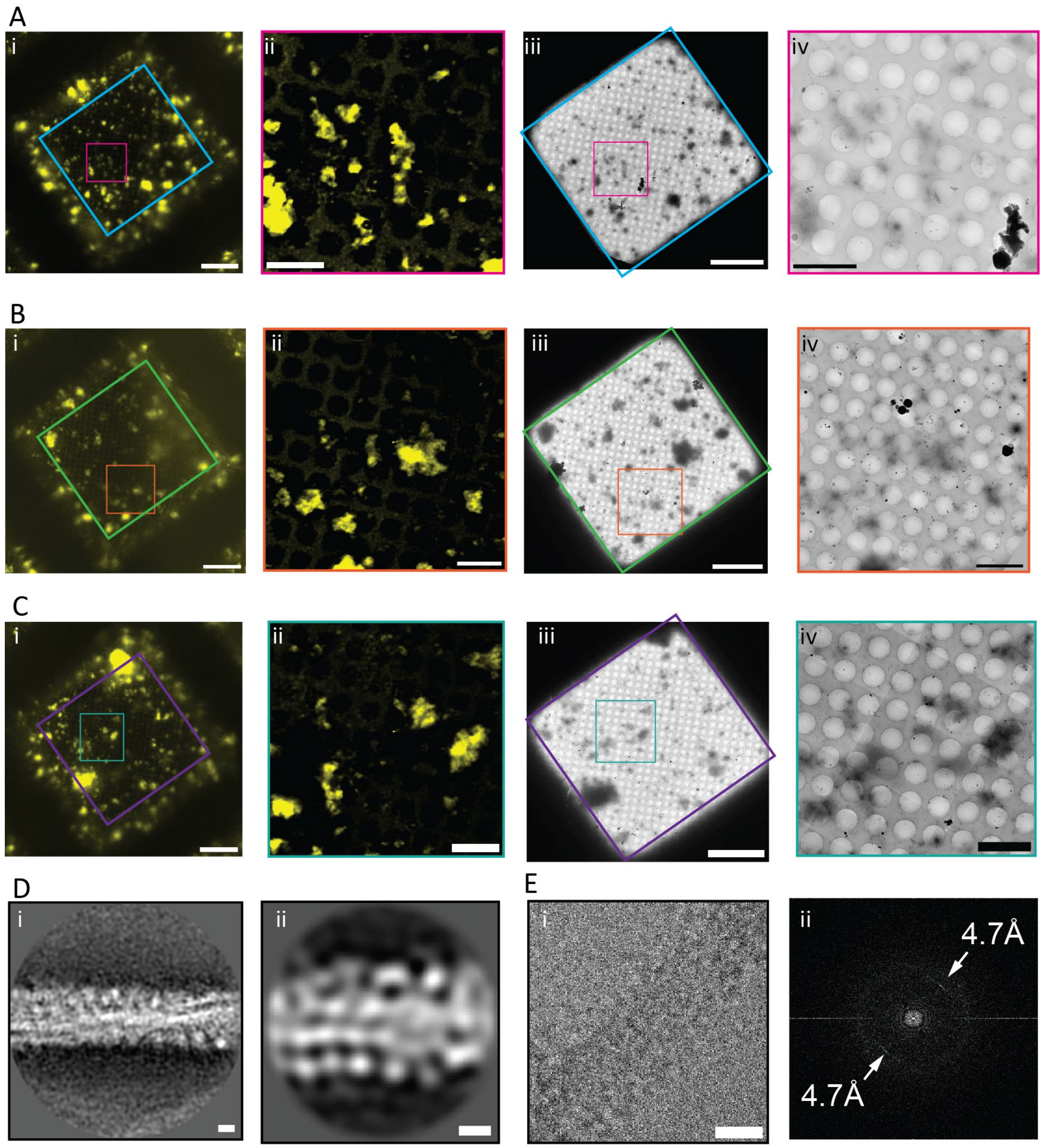
A
i
ii
iii
iv
B
i
ii
iii
iv
C
i
ii
iii
iv
D
i
ii
E
i
ii
4.7Å
4.7Å

Sup. Fig. 1. Correlative cryo-light and electron microscopy of *ex vivo* tau-YFP aggregates. (A-C) Additional examples showing the correlation between the YFP fluorescence (i, ii) and the electron density (iii, iv) of the tau-YFP filamentous assemblies. Images obtained by cryo-confocal microscopy (i,ii) and cryo-electron microscopy (iii, iv), respectively. Scale bars: 20 µm (i, iii), 5 µm (ii, iv). (D) 2D class averages of YFP-tagged tau amyloid fibrils. When the particles were finely aligned (i), the fibril twist (~650 Å crossover) was clearly recognizable whereas the YFP decoration was dim. When the particles were coarsely aligned, with less accuracy with respect to one another (ii), bright dots densely decorated the tau amyloid fibril and could be distinguished from each other in the 2D class average. (E) A representative micrograph with a filament along the diagonal (i) and its corresponding Fast Fourier Transform (FFT, ii). A peak at 4.7 Å, which is a feature of amyloid structure, is highlighted in the FFT.

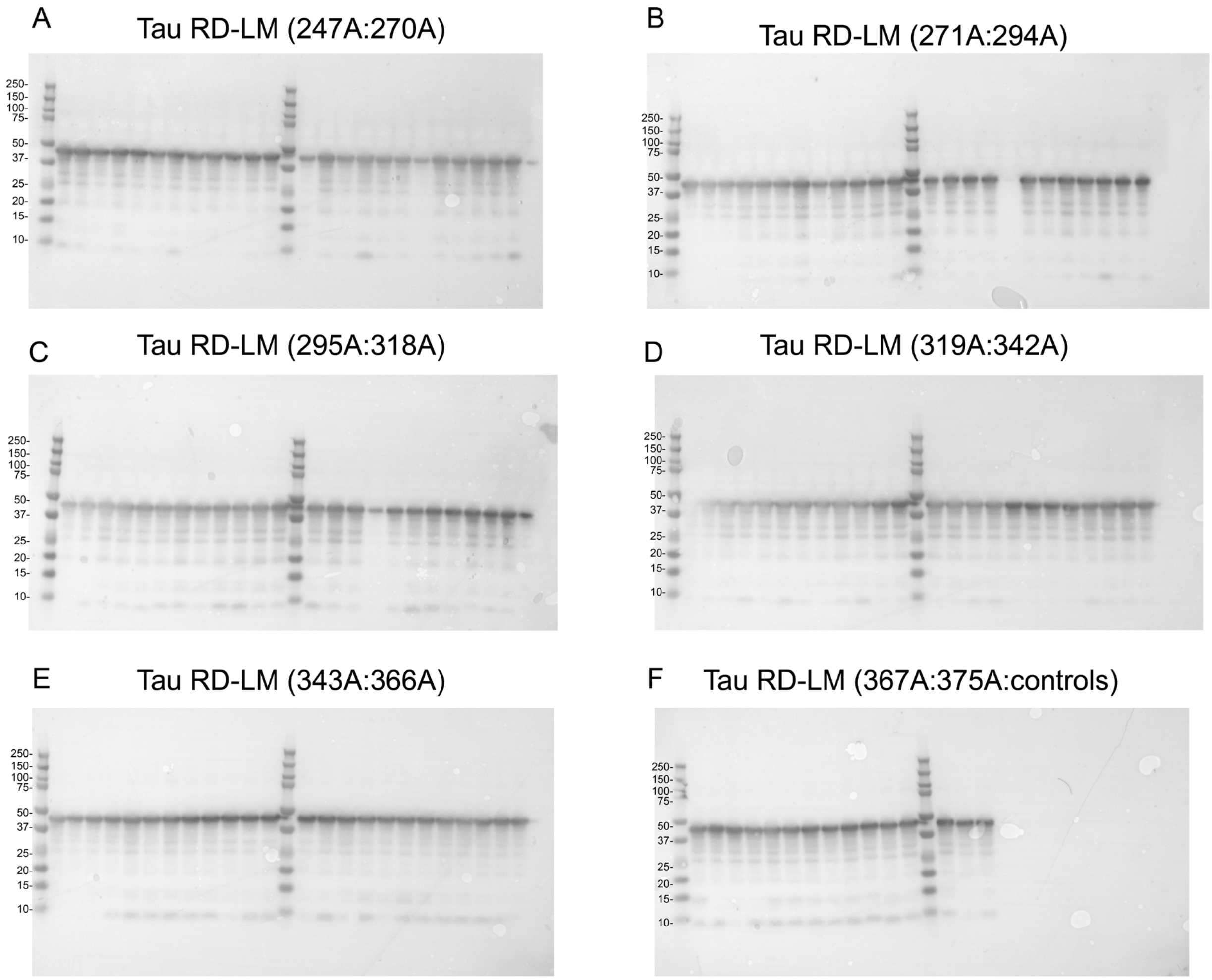
A
Tau RD-LM (247A:270A)
B
Tau RD-LM (271A:294A)
C
Tau RD-LM (295A:318A)
D
Tau RD-LM (319A:342A)
E
Tau RD-LM (343A:366A)
F
Tau RD-LM (367A:375A:controls)
250-
150-
100-
75-
50-
37-
25-
20-
15-
10-

Sup. Fig. 2 Validation of intact protein expression of all mutants and control. Western blots of 10µg of clarified cell lysate from HEK293T cells expressing each alanine variant demonstrated robust expression of fusion protein. Lanes were loaded with each variant in consecutive order. Tau protein was detected using an in-house rabbit polyclonal antibody, named Tau A, which targets R1.

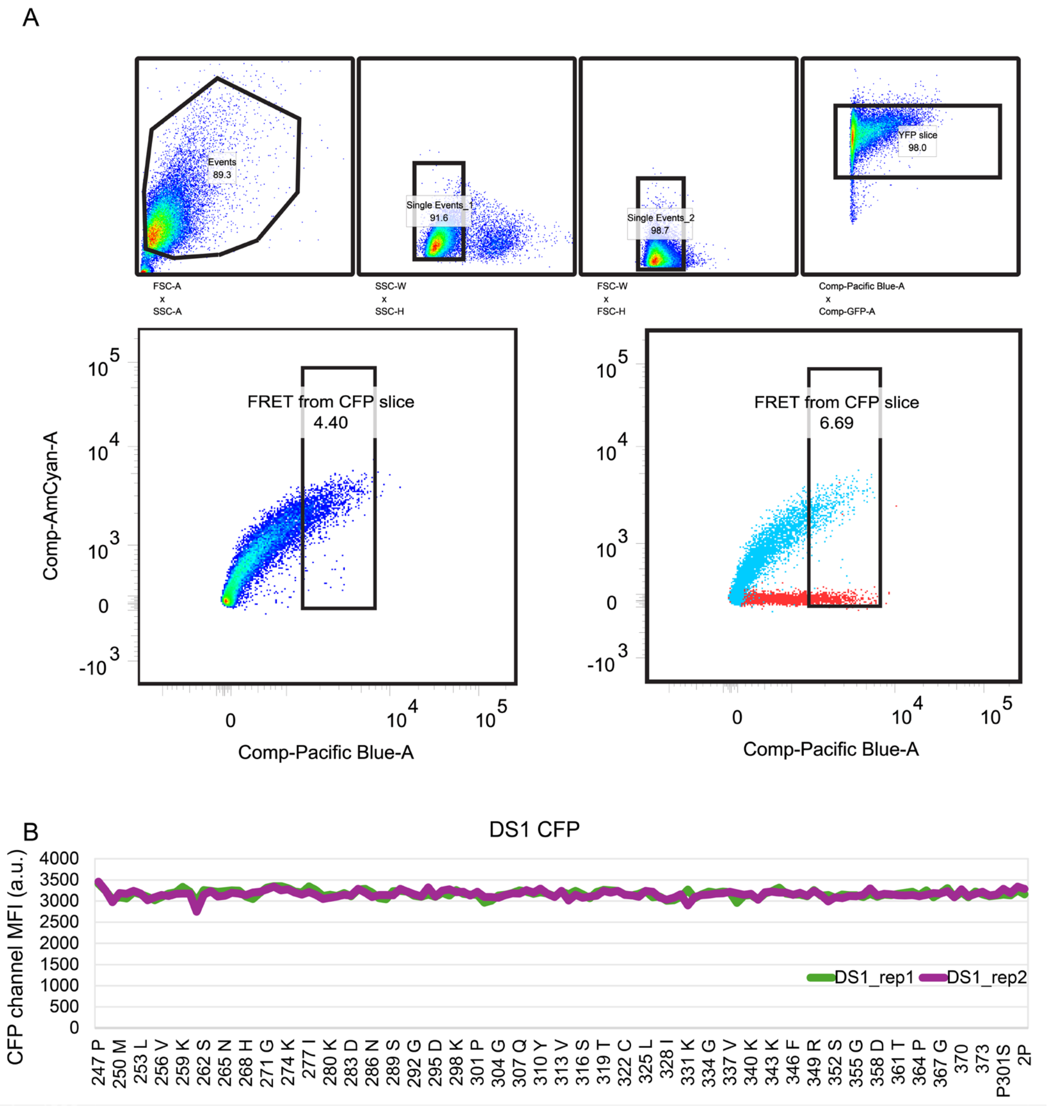

A
Events
89.3
FSC-A
x
SSC-A
Single Events_1
91.6
SSC-W
x
SSC-H
Single Events_2
98.7
FSC-W
x
FSC-H
YFP slice
98.0
Comp-Pacific Blue-A
x
Comp-GFP-A
Comp-AmCyan-A
FRET from CFP slice
4.40
FRET from CFP slice
6.69
Comp-Pacific Blue-A
Comp-Pacific Blue-A
B
DS1 CFP
CFP channel MFI (a.u.)
4000
3500
3000
2500
2000
1500
1000
500
0
DS1_rep1
DS1_rep2
247 P
250 M
253 L
256 V
259 K
262 S
265 N
268 H
271 G
274 K
277 I
280 K
283 D
286 N
289 S
292 G
295 D
298 K
301 P
304 G
307 Q
310 Y
313 V
316 S
319 T
322 C
325 L
328 I
331 K
334 G
337 V
340 K
343 K
346 F
349 R
352 S
355 G
358 D
361 T
364 P
367 G
370
373
P301S
2P

Sup. Fig. 3. Gating strategy for LM Ala scan. Events consistent with size and distribution of HEK293 cells were selected for analysis. These were gated for homogenous side-scattering and forward scattering. Then a slice through the center of the YFP (GFP channel) population was selected to measure cells with similar levels of YFP expression. Last the median fluorescence intensity of the FRET channel (AmCyan) through a slice of high CFP intensity (Pacific Blue channel) was recorded. In blue, an example of the gate used to quantify the MFI of a positive control Tau RD(LM)-CFP. An anti-aggregation negative control, Tau RD(LM/2P)-CFP, is shown in red to demonstrate the degree of signal to noise. (B) The median fluorescent intensity of CFP measured in the Pacific Blue channel of the gated population showed relatively homogeneous levels of expression of all variants.

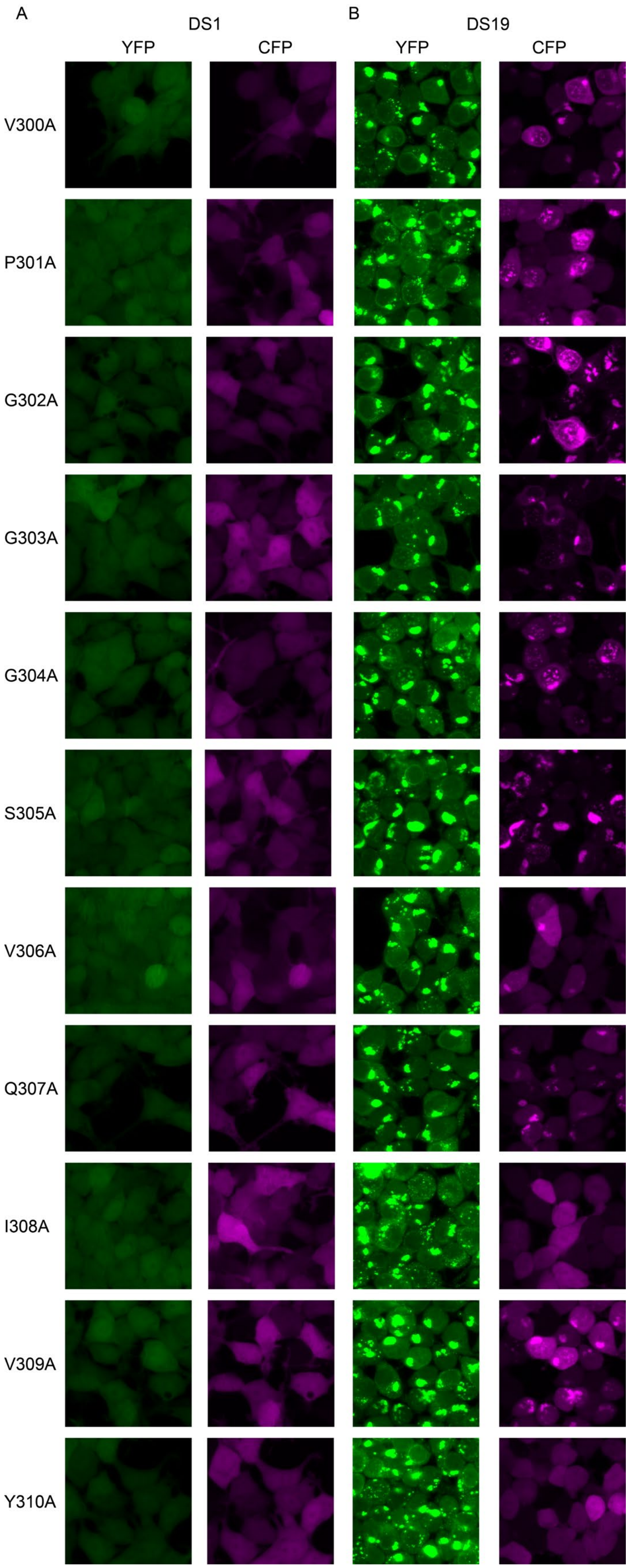

A
DS1
YFP
CFP
B
DS19
YFP
CFP
V300A
P301A
G302A
G303A
G304A
S305A
V306A
Q307A
I308A
V309A
Y310A

Sup. Fig. 4. (LM)-CFP inclusions co-occur with (LM)-YFP aggregates. Confocal microscopy of selected Tau RD (LM)-CFP variants 48 hours after lentiviral transduction of DS1 (A) and DS19 cells (B). Importantly, no inclusions were observed in DS1 cells which lack (LM)-YFP aggregates, while (LM)-CFP inclusions became apparent in cells with (LM)-YFP inclusions. Notably, not all variants contained (LM)-CFP inclusions (e.g. I308A and Y310A).

A

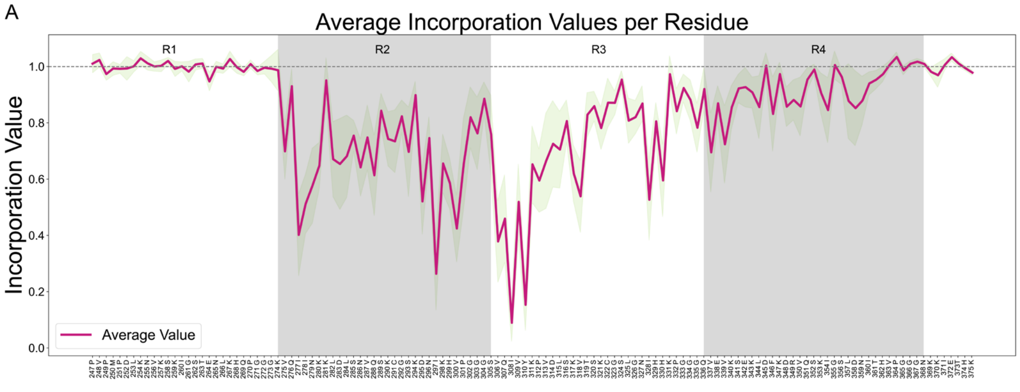
Average Incorporation Values per Residue
R1
R2
R3
R4
Incorporation Value
1.0
0.8
0.6
0.4
0.2
0.0
Average Value

Sup. Fig. 5. Average Ala scan of all DS strains. (A) Line plot of the average incorporation values for all strains revealed a distributed region that spans R2, R3 and R4 and was involved in aggregation in cells. Shaded area represents 95% confidence interval. Importantly, usage of the ^{306}VQIVYK^{311} region was a conserved feature among all strains. Throughout the length of the repeat domain, we found regions with patterns that involved every other residue, reminiscent of the expected alternating patterns in the beta-strands of an amyloid, for example: ^{293}SDNIKHV^{300}, ^{305}SVQIVYKPVDLSK^{317}and ^{326} GNIHHKP^{332}.

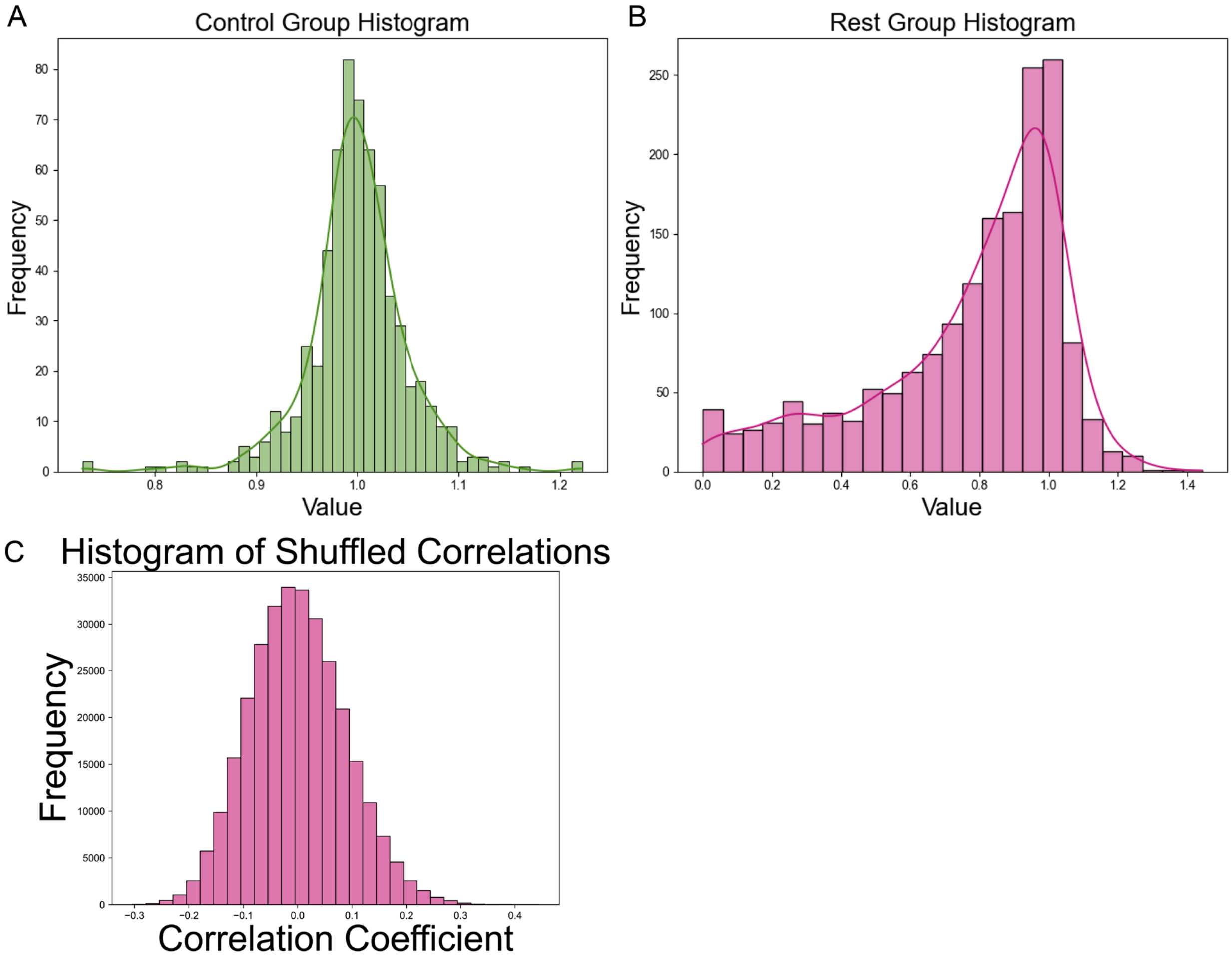
A
Control Group Histogram
Frequency
Value
B
Rest Group Histogram
Frequency
Value
C
Histogram of Shuffled Correlations
Frequency
Correlation Coefficient

Sup. Fig. 6. Shuffled correlations for strain and residue. To test the ability of the Ala scan to determine covariance of the scanned signature between strains, we considered the domains of tau RD that were not affected by Ala substitution (Control group: refers to non-variant residues, that is, the first 20 and last 10 amino acids of the sequence), and those that were (Rest Group: refers to the remaining residues). (A)The Control group values were randomly distributed around 1 in terms of their effect on incorporation. (B) The remaining sequences were highly skewed in their distribution, indicating that the population of hits were non-randomly distributed. (C) To test to what degree the observed correlations were meaningful, for each strain we generated 1000 permutations of independently shuffled values for each residue. This bounds the spurious correlations at an R^2 of 0.3, which is much lower than most of the observed correlations in Fig.4A.

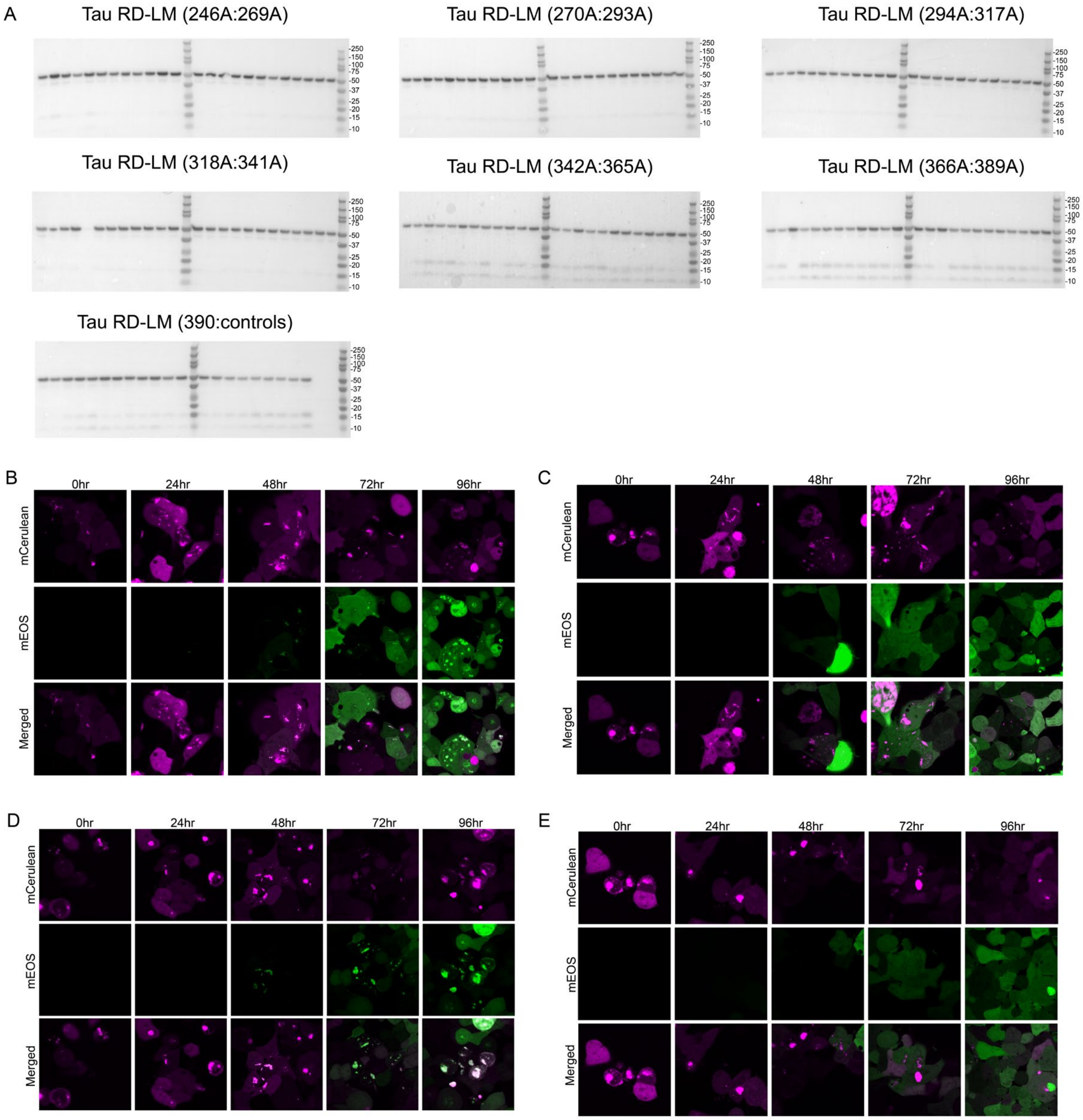

A
Tau RD-LM (246A:269A)
Tau RD-LM (270A:293A)
Tau RD-LM (294A:317A)
Tau RD-LM (318A:341A)
Tau RD-LM (342A:365A)
Tau RD-LM (366A:389A)
Tau RD-LM (390:controls)
-250
-150
-100
-75
-50
-37
-25
-20
-15
-10
B
C
D
E
0hr
24hr
48hr
72hr
96hr
mCerulean
mEOS
Merged

Sup. Fig. 7. WT Ala scan validation. (A) Western blots of 10μg of clarified cell lysate from HEK293T cells treated with all Tau RD(4Rext)-Cer alanine variants demonstrated robust expression of the fusion protein. Lanes were loaded with each variant in consecutive order. Tau protein was detected using an in-house rabbit polyclonal antibody, named Tau A, which targets R1. Confocal images of incorporation time course in: (B) AD seeded RD(3R/4Rext)-Cer/Ruby cells treated with WT-tau 246-408-mEOS3.2 (C) AD seeded RD(3R/4Rext)-Cer/Rub cells treated with 2P-tau 246-408-mEOS3.2. (D) AD seeded RD(4Rext)-Cer/Ruby cells treated with WT-tau 246-408-mEOS3.2. (E) AD seeded RD(4Rext)-Cer/Rub cells treated with 2P-tau 246-408-mEOS3.2. Importantly, the addition of the two proline mutations that inhibit tau's ability to aggregate prevented inclusion formation in cells. No puncta formed even after 96hrs of expression in cells that contained aggregates. Please note that the 96-hour timepoints for all four conditions were also used in Figure 6 Panel B.

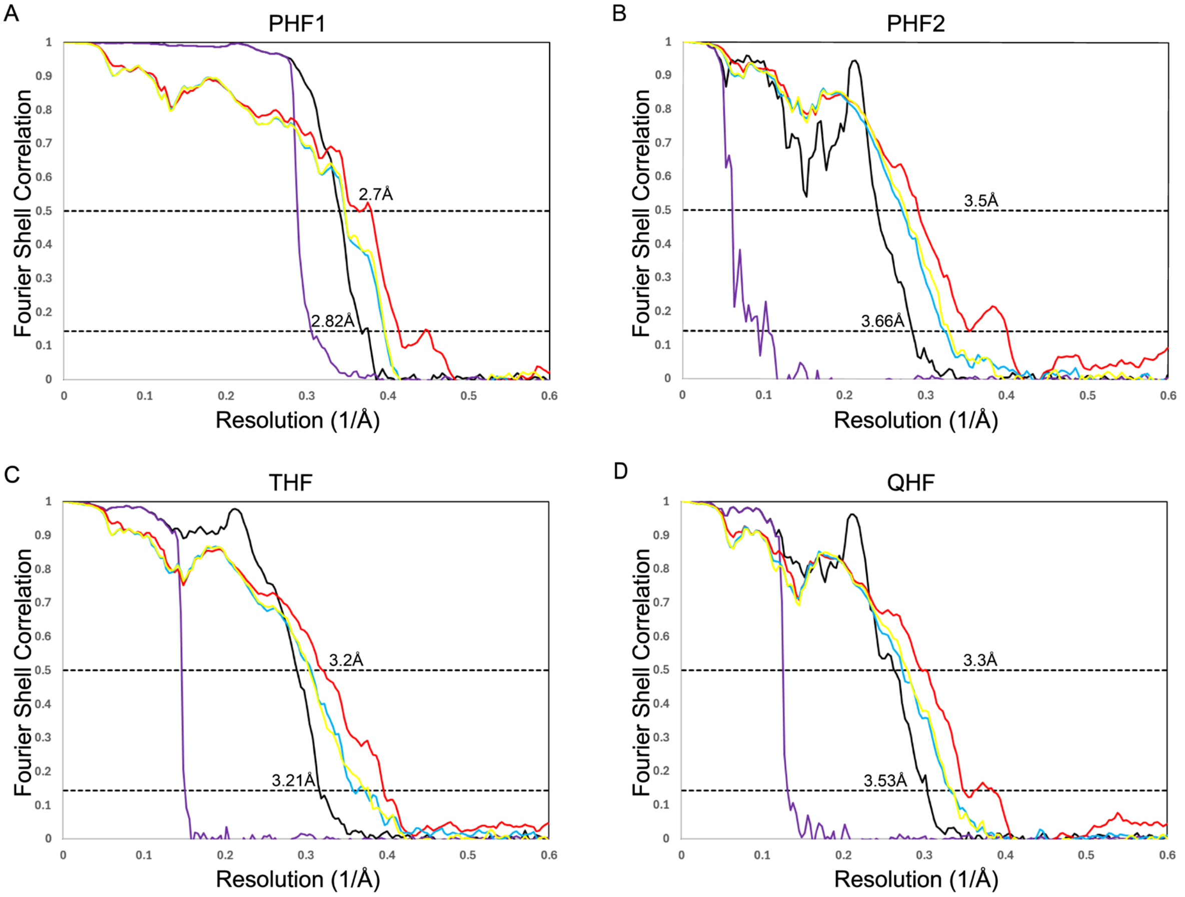

E

Filament	Chain	RMSD to chain A of PDB:5o3L (Angstrom)
PHF1	A	0.897
PHF1	B	0.902
PHF2	H	1.32
PHF2	I	1.376
THF	L	1.191
THF	I	2.541
THF	F	1.482
QHF	A	1.669
QHF	B	1.285
QHF	K	1.339
QHF	L	1.734

F

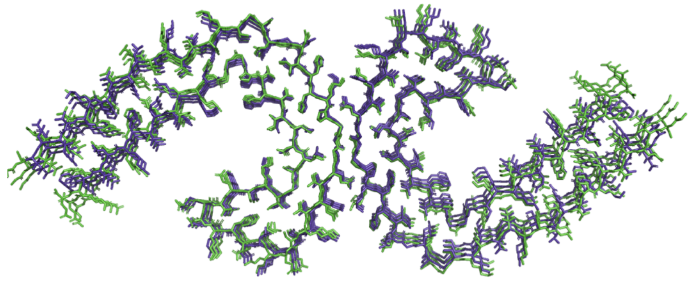

Sup. Fig. 8. CryoEM reconstructions and atomic modeling. Graphs illustrate the Fourier Shell Correlation (FSC) curves for the cryoEM models of tau (287-391) derived PHF1 (A), PHF2 (B), THF (C) and QHF (D), shown in black in each plot. Additionally, FSC curves are presented for the refined atomic model against the post-processed map utilized for atomic model generation (Red), for the refined atomic model from one half-map to itself (Blue), for the refined atomic model from the first half-map to the second (Yellow), and for phase randomized curves of the two independently refined half-maps (Purple). (E) Table showing the root mean-squared deviation of a single rung of each protofilament from the atomic models of tau (287-391) when compared to chain A of PDB 5o3l, which has the AD fold. Overlay of 3 rungs of PDB5o3l (purple) with 3 rungs of PHF1(green).

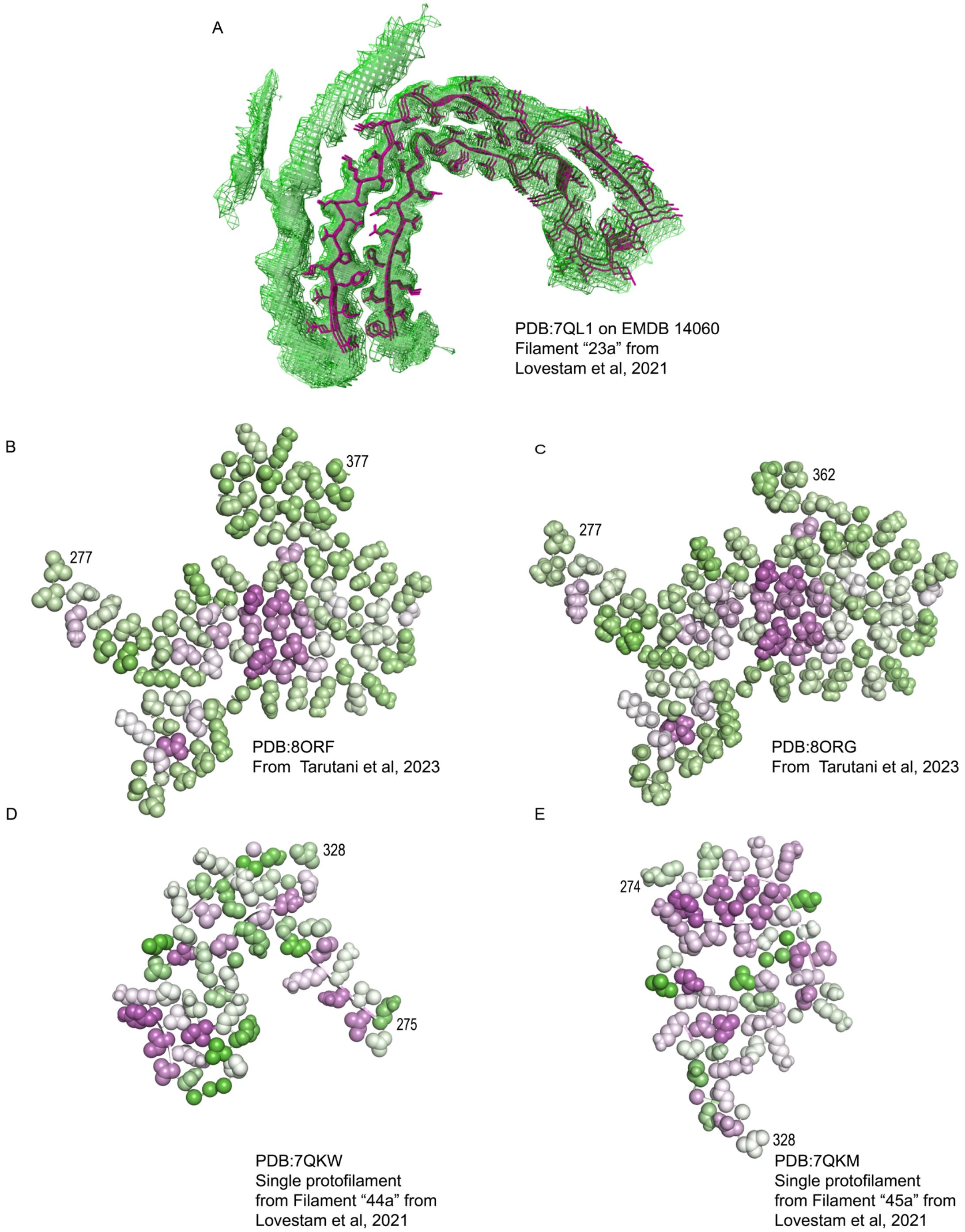
A
PDB:7QL1 on EMDB 14060
Filament "23a" from
Lovestam et al, 2021
B
377
277
PDB:8ORF
From Tarutani et al, 2023
C
362
277
PDB:8ORG
From Tarutani et al, 2023
D
328
275
PDB:7QKW
Single protofilament
from Filament "44a" from
Lovestam et al, 2021
E
274
328
PDB:7QKM
Single protofilament
from Filament "45a" from
Lovestam et al, 2021

Sup. Fig. 9. Positioning of Ala hits in relation to structures. (A) *In vitro* assembled filaments made from tau (266-391) in PBS in the CTE fold, modeled in PDB:7QL1 into the density from EMDB 14060. The density threshold was set to highlight extra unmodeled density appearing to wrap back onto the core, presumably composed of the R2 sequence. (B and C). Two polymorphs reconstructed from filaments extracted from SH-S5Y5 cells expressing 1N4R tau and seeded with CBD brain homogenate. They were colored by the incorporation values measured for the average of all CBD cases. (D and E) PDB 7QKW of filament “44a” assembled from recombinant tau (266-391 S356D) in 10mM PB Ph7.4, 10mM DTT and 200mM KCl and PDB 7QKM of filament “45a” assembled from recombinant tau (266-391 S356D) in 10mM PB Ph7.4, 10mM DTT and 200mM NaCl. Both filament types were obtained in our preparation of Tau (266-391) in PBS with 10mM DTT (as in condition 23a from Lövestam et. al, 2022), importantly we did not observe filaments with the CTE fold and instead observed “44a” and “45a” in the absence of the S356D mutation. Both were colored by the incorporation values obtained from the preparation with these mixed filaments. Purple represents low incorporation and green represents no change in incorporation.

Table S1. DS strain library

Strain	Inclusion Appearance	Origin
2	Mosaic	AGD cell line
3	Ordered	Recombinant Fibrils
4	Speckles	AD Homogenate
5	Speckles	Recombinant Fibrils
6	Threads	P301S Mouse Homogenate
7	Speckles	Recombinant Fibrils
8	Speckles	P301S Mouse Homogenate
9	Speckles	Recombinant Fibrils
10	Ordered	Recombinant Fibrils
11	Disordered	CBD Cell line
12	Speckles	CBD Cell line
13	Disordered	CBD Cell line
14	Ordered	Recombinant Fibrils
15	Threads	Recombinant Fibrils
16	Speckles	CBD Cell line
17	Speckles	AD cell line
18	Speckles	CTE Homogenate
19	Ordered	Recombinant Fibrils

Table S2. Subjects studied

Sample Name	ID	Age	Sex	Institution
AD1	49341	83	F	UTSW
AD2	46879	76	M	UTSW
AD3	46121	74	F	UTSW
AD5	45408	73	M	UTSW
62579_Parietal	62579	71	M	WashU
63377_Parietal	63377	86	M	WashU
63870_Parietal	63870	81	F	WashU
63870_Temporal	63870	81	F	WashU
CBD_12041	12044	73	M	WashU
CBD1	48638	64	M	UTSW
CBD2	44193	55	M	UTSW
CBD5	49231	74	M	UTSW
CBD6	46267	67	F	UTSW
CBD7	37872	68	M	UTSW
CBD_3950	3950	72	M	WashU
CBD_23302	22302	62	M	WashU
CTE_9130	9130			Boston University
CTE_8408	8408			Boston University
PSP2	45460	68	F	UTSW
PSP1	47941	69	M	UTSW
PSP3	49017	61	F	UTSW
PSP4	48221	67	M	UTSW
PSP_F1	A06-231	76	F	WashU

Table S3. CryoEM data collection and refinement statistics

Cryo-EM data collection, refinement, and validation statistics	Truncated Tau 287-391 w/ 200mM MgCl2				Truncated Tau 297-391 w/ 200mM MgCl2			Truncated Tau 266-391 w/ 200mM NaCl	
Electron Microscope Type	Titan Krios				Titan Krios			Titan Krios	
Nominal Magnification	105,000x				105,000x			105,000x	
Voltage (kV)	300				300			300	
Detector	Gatan K3 w/ Bioquantum Energy Filter				Gatan K3 w/ Bioquantum Energy Filter			Gatan K3 w/ Bioquantum Energy Filter	
Electron Exposure (e-/Å²)	57				52			62	
Defocus Range (μm)	-0.8 to -2.2				-1.0 to -2.2			-1.5 to -3.0	
Super-resolution Pixel Size (Å)	0.415				0.415			0.415	
**Reconstruction**	**PHF**	**THF**	**QHF**	**SF**	**PHF**	**THF**	**QHF**	**44a**	**45a**
Total number of micrographs									
Number of usable micrographs	2,277				5,094			2,528	
Particles after Extraction	336,719				288,528			348,124	
Particles after 2D Classification	163,219							142,881	137,756
Particles after 3D Classification	152,026	41,201	20,671	16,801	50,023	28,122	80,078		
Map Resolution (Å, FSC=0.143)	2.82	3.21	3.53	3.66	3.21	3.32	2.79	4.41	3.86
Helical Rise (Å)	2.37	4.75	2.37	4.75	2.38	4.75	4.75	4.82	2.41
Helical Twist (°)	179.47	-0.86	179.58	-0.87	179.5	-0.86	-0.81	-0.93	179.58
Symmetry Imposed	2-1	C1	2-1	C1	2-1	C1	C1	C2	C1
EMDB Entry	EMD-46908	EMD-46911	EMD-46915	EMD-46909	EMD-46870	EMD-46885	EMD-46904	EMD-47092	EMD-46937
**Refinement**									
Initial model used (PDB Code)	5o3l	5o3l	5o3l	5o3l				7QKW	7QKM
Model resolution (Å, FSC=0.5)	2.7	3.2	3.3	3.5					
Map sharpening B factor (Å²)	-52.32	-62.94	-59.15	-65.17					
Model composition									
Non-hydrogen atoms	4,419	6,625	14,371	4,416					
Protein residues	462	693	924	462					
Ligands	N/A	N/A	N/A	N/A					
B Factors (Å²)									
Protein	72.95	72.59	72.95	72.95					
Ligand	N/A	N/A	N/A	N/A					
R.m.s. deviations									
Bond lengths (Å)	0.010 (0)	0.011 (0)	0.011 (0)	0.011 (0)					
Bond angles (°)	1.941 (20)	2.024 (40)	1.982 (37)	2.056 (26)					
Validation									
MolProbity score	0.95	1.12	1.15	1.41					
Clashscore	0	0	0.07	0					
Poor rotamers (%)	0	1.16	0.75	1.74					
Ramachandran plot									
Favored (%)	92.89	89.33	86.78	80.44					
Allowed (%)	7.11	10.67	13.22	19.56					
Disallowed (%)	0	0	0	0					

Other Supplementary Materials for this manuscript include the following:

Movies S1: **cryo-ET of DS13-extracted filaments**

Data S1: **Source data.**
